# Immune responses to alglucosidase in infantile Pompe disease: recommendations from an Italian pediatric expert panel

**DOI:** 10.1186/s13052-022-01219-4

**Published:** 2022-03-05

**Authors:** Vincenza Gragnaniello, Federica Deodato, Serena Gasperini, Maria Alice Donati, Clementina Canessa, Simona Fecarotta, Antonia Pascarella, Giuseppe Spadaro, Daniela Concolino, Alberto Burlina, Giancarlo Parenti, Pietro Strisciuglio, Agata Fiumara, Roberto Della Casa

**Affiliations:** 1 Division of Inherited Metabolic Diseases - Department of Diagnostic Services, University Hospital of Padua, Padua, Italy; 2 Department of Translational Medical Sciences, University of Naples Federico II, Naples, Italy; 3 Unit of Metabolic Diseases, Ospedale Pediatrico Bambino Gesù, IRCCS|, Rome, Italy; 4 Metabolic Rare diseases Unit - Pediatric Department, Fondazione MBBM, University of Milano-Bicocca, Monza, Italy; 5 Metabolic and Neuromuscular Unit, Meyer Children Hospital-University of Florence, Florence, Italy; 6 Division of Pediatric Immunology - Department of Health Sciences, Meyer Children Hospital-University of Florence, Florence, Italy; 7 AORN Santobono Pausilipon, Naples, Italy; 8 Pediatrics - Science of Health Department, University “Magna Graecia”, Catanzaro, Italy; 9 Telethon Institute of Genetics and Medicine, Naples, Italy; 10 Regional Referral Centre for Metabolic Diseases (CRR-MET), UOC Pediatric Clinic - Department of Clinical and Experimental Medicine, University of Catania, Catania, Italy; 11 Dipartimento Materno Infantile, A.O. San Pio, Benevento, Italy

**Keywords:** Pompe disease, CRIM status, Immune tolerance induction, Infusion associated reactions, Desensitization

## Abstract

**Background:**

Classic infantile onset of Pompe disease (c-IOPD) leads to hypotonia and hypertrophic cardiomyopathy within the first days to weeks of life and, without treatment, patients die of cardiorespiratory failure in their first 1–2 years of life. Enzymatic replacement therapy (ERT) with alglucosidase alfa is the only available treatment, but adverse immune reactions can reduce ERT’s effectiveness and safety. It is therefore very important to identify strategies to prevent and manage these complications. Several articles have been written on this disease over the last 10 years, but no univocal indications have been established.

**Methods:**

Our study presents a review of the current literature on management of immune responses to ERT in c-IOPD as considered by an Italian study group of pediatric metabolists and immunologists in light of our shared patient experience.

**Results:**

We summarize the protocols for the management of adverse reactions to ERT, analyzing their advantages and disadvantages, and provide expert recommendations for their optimal management, to the best of current knowledge. However, further studies are needed to improve actual management protocols, which still have several limitations.

## Introduction

Pompe disease is an autosomal recessive lysosomal storage disorder in which deficiency of the acid α-glucosidase (GAA) results in a build-up of glycogen in multiple tissues, particularly in the cardiac, skeletal and smooth muscle tissues [1]. Classic infantile onset of Pompe disease (c-IOPD), its most severe form, presents in the first days to weeks of life with hypotonia and hypertrophic cardiomyopathy. Without treatment, patients die of cardiorespiratory failure within the first 1–2 years of life [2–4]. Enzymatic replacement therapy (ERT) with alglucosidase alfa (rh-GAA) is the only known treatment available [5]. Recommended dosage is 20 mg/kg every other week, although recent studies have reported that patients receiving up to 40 mg/kg/week have better outcomes [6–8]. ERT has been shown to improve ventilator-free and overall survival as well as cardiomyopathy and motor functions [9–11]. However, immune reactions can reduce ERT’s effectiveness and safety, thereby raising an important issue [12, 13].

## Materials and methods

An Italian Pediatric study group on immune response to ERT in patients with Pompe disease consisting of pediatric metabolists and immunologists was established in December 2018 under the patronage of the Italian Society for Metabolic Diseases and Neonatal Screening (SIMMESN). The group’s overall objectives were to assess the impact of immune reactions to ERT as measured by efficacy and safety and provide an expert panel consensus on the modalities, risks and benefits of immunomodulating and desensitizing therapy, particularly in patients with c-IOPD. We analyzed studies published from 2000 to 2021. They were identified by searching Pubmed, Medline and Embase, using the search terms “Pompe disease AND immune response”, “Pompe disease AND immunomodulation”, “Pompe disease AND hypersensitivity reactions”, “Pompe disease AND desensitization”. The literature search was supplemented by reviewing relevant citations in the in initial article identified. We screened title/abstract, reviewed full texts and extracted data. Exclusion criteria were studies published in language other than English, unpublished studies and abstracts. This study is a summary of the evidence present in the literature as a basis of discussion based upon direct patient experience concluding with expert opinions and recommendations for the optimal management of IOPD patients.

## Results and discussion

### Types of immune reactions to ERT

Two types of immune reactions against ERT have been reported: 1) infusion associated reactions (IARs) including hypersensitivity responses with or without increase of specific immunoglobulin E (IgE) and 2) development of specific immunoglobulin G (IgG) which reduces treatment efficacy by way of two different mechanisms: a) uptake of the administered enzyme in Fc receptor expressing cells such as monocytes and macrophages (binding, non-neutralizing antibodies) and b) targeting functional or catalytic domains (neutralizing antibodies) of the replaced enzyme [12, 14].

Antibodies and antigens can also form immune complexes and trigger a cascade of adverse events [15]. In addition to an antibody response, ERT has also been shown to induce a T cell response [16].

Based on anti-rhGAA IgG antibody titers, patients are classified into 3 groups [17]:

- HSAT (high and sustained antibody titer): ≥51,200 on ≥2 occasions at or beyond 6 months on ERT
- SIT (sustained intermediate titer): ≥12,800 and < 51,200 within the first year of ERT
- LT (low titer): < 6400 within the first year of ERT.

HSAT is closely associated with clinical decline [18]. Moreover, there is evidence that antibodies levels ≥12,800 (lower range of SIT) over a certain period of time also reduce the enzyme’s efficacy [5]. It appears that the persistence of sustained titers for a long period of time, rather than the absolute values of the titers, impairs the ERT efficacy and thus the clinical outcome [19]. Approximately 90% of IOPD patients treated with alglucosidase alfa develop IgG antibodies [5, 20], with a large majority developing immunological tolerance with continued treatment [18, 21]. Most pediatric patients develop antibodies in the first 3 months of therapy, but this is not always the case [11]. Sometimes, antibodies become positive or peak several years after beginning ERT [22].

- **Statement #1**: On the basis of literature data and the personal experience of the authors, the Expert Panel suggests IgG assay at time 0, monthly for 12 months, and every 3 months thereafter. After many years, IgG assay is indicated only in cases of clinical deterioration and always in cases of adverse reactions [23]. If positive, the sample must be further tested for neutralizing antibodies [12]. IgE is measured in the setting of a hypersensitivity reaction and is not routinely tested [24]. Assays available include non-standard laboratory tests and are carried out by specialized labs or by the drug manufacturer’s producer.

### Risk factors for immunogenicity: CRIM (cross reactive immunological material) status

Several factors need to be considered in assessing immunogenicity risk. Index signs of the severity of the clinical phenotype such as early onset of symptoms, severe cardiomyopathy, and residual enzyme activity (< 1%), have been considered by some authors to be risk factors for the development of a high antibody titer, while there seems to be no link with ERT dose, infusion rate, and age at the time of therapy inception, contrary to what was assumed in the past [12, 22]. The most important factor indicated appears to be CRIM status [20, 25, 26].

Patients with no detectable GAA protein are classified as CRIM negative (CN), so that their immune system fails to tolerate ERT and recognizes it as foreign (about 30% of patients with c-IOPD). Patients with some residual GAA protein (active or inactive forms) are classified as CRIM positive (CP), and usually do not mount an immune response or else mount a transient low titer response [20, 25]. Previous studies have demonstrated that in CRIM negative patients antibody titers were higher, seroconversion occurred earlier (by 4 weeks), titers were sustained at high levels, and neutralizing antibodies often developed. Conversely, CRIM positive patients showed a variable time to seroconversion (4–64 weeks, median 8 weeks) with either no antibody response or a non-neutralizing antibody response with a low peak titer that diminished with continued therapy [20, 26].

Serotiter levels play a role in the clinical decline of the CRIM negative patients. Kishnani et al. reported that both CRIM positive and CRIM negative groups showed similar cardiological status (left ventricular mass index), gross motor development, and biomarker levels (eg tetrasaccharide) at baseline. After 26 weeks of ERT both groups showed improvement but at 52 weeks, CRIM positive patients demonstrated additional improvement, while the CRIM negative group worsened in correlation with the increased and persistent antibody response. By 27.1 months of age, all CRIM negative patients were deceased or on ventilators compared to 19% of the CRIM positive patients [20]. Similar results were reported by Van Gelder et al. [27].

A previous Italian experience on 28 patients, followed for a median period of 6 years, also demonstrated that, compared to CRIM negative, CRIM positive patients showed a better outcome. In particular, the risk of death in CRIM positive patients was 1/4 and ventilator free survival risk was 1/5 compared to CRIM negative patients. CRIM positive patients also showed improved motor and cardiac outcomes. Of note, data concerning the titers of anti-rhGAA antibodies were available in 13 patients (7 CRIM positive, 5 CRIM negative, 1 unknown) and all CRIM positive patients have no antibodies or were LT, while all CRIM negative patients were SIT or HSAT [28].

Immune tolerance induction (ITI) protocols can be used for prophylaxis, to preempt immune response in ERT naive patients (prophylactic approach), and for therapy, to decrease existing antibodies in ERT treated patients with previously established immune responses. For this purpose, it is important to know CRIM status before starting ERT. The gold standard to determining CRIM status is the western blot analysis of skin fibroblast lysates. However, skin biopsies are invasive and require several weeks. A more rapid method for determining CRIM status using western blot analysis of blood mononuclear cells (PBMCs) was successfully tested on a small number of patients (*n* = 8) [29] but its sensitivity was assessed at only around 82% in a subsequent study including 33 patients [30]. CRIM status can also be predicted based on GAA gene mutations in more than 90% of patients. The types of mutations in Pompe disease are shown in Table 1 [31, 32]. If, based on the mutation, CRIM status is not already foreseen, a western blot on fibroblasts should be performed [31].

**Fig. 1:**
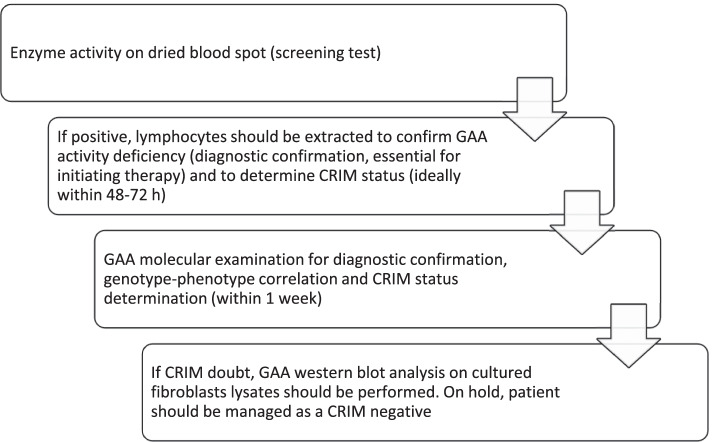
Approach to a child with suspected Pompe disease

- **Statement #2:** Before starting ERT in a c-IOPD patient, it is essential to determine the CRIM status. The protocol recommended by the Expert panel is shown in Fig. 1. This protocol provides information useful for therapeutic decisions in about 1 week.

**Table 1:** Types of mutations in Pompe disease [31]

Type of mutation	Effect	Exceptions
Nonsense, frameshift, multiple exon deletion mutations	Two alleles: CRIM negative	-Premature termination codon in the last exon or up to above 50 nucleotides from the 3′ end of the penultimate exon - In frame deletion
Missense mutation	At least one: CRIM positive	Point mutation that abolished the initiator methionine codon
Splicing mutation (about 15%)	Difficult to predict	–

*CRIM* Cross-reactive immunologic material

### Prophylaxis protocols in CRIM negative patients

Most CRIM negative patients develop HSAT, but limited information is available to accurately predict those who are least likely to develop it. Accordingly, a risk-benefit ratio supports initiating an immunotolerance protocol at the time of ERT inception. Advantages of preemptive immunotolerance induction are exposure to fewer drugs, shorter duration of therapy and improved clinical outcomes by preventing prolonged exposure to HSAT [33]. Several protocols have been used as described below and summarized in Table 2.

**Table 2:** Prophylaxis protocols in naive patients

**CRIM negative patients**	
*RTX + MTX+ IVIG (short course, 5 weeks)* [23, 34, 35]^a^	
• RTX 375 mg/m2 IV (or if body surface area < 0.5 m^2^ = 12.5 mg/kg) weekly four times, the first dose given 1 day before the first ERT administration.	
• MTX 0.4 mg/kg sc/orally, 3 doses per week or MTX 1 mg/kg/weekly [36] for almost 3 weeks with or without IVIG 400–500 mg/kg monthly for a period of 5–6 months (until B cell levels had reached normal values for age)	
*RTX + sirolimus or mycophenolate + IVIG* [37]	
• RTX IV: 750 mg/m^2^ 10–14 days apart or 375 mg/m^2^ per week for 3 weeks (dosed depending on the infant’s clinical status and ability to tolerate IV fluids).	
• Sirolimus 0.6–1 mg/m^2^ per day adjusted to maintain serum level of 3–7 ng/ml or mycophenolate 300 mg/m^2^ per day.	
• IVIG 500–1000 mg/kg adjusted to maintain serum IgG levels of 700–1000 mg/dl.	
• After an initial pre-ERT course of immunomodulation (3 weeks), ERT is initiated alongside maintenance with every 12-week RTX, daily sirolimus or mycophenolate mofetil and monthly IVIG administration.	
**CRIM positive patients**	
*Low-dose MTX* [17]	
• MTX at 0.4 mg/kg body weight is administered on the day of ERT infusion subcutaneously (15 min before or orally 1 h before if subcutaneous administration is not possible) and again on the following 2 days with the first 3 ERT infusions.	
*RTX + MTX+ IVIG (short course, 5 weeks)* [38]	
• See above (CRIM negative patients)	

*CRIM* cross-reactive immunologic material, *ERT* enzyme replacement therapy, *RTX* rituximab, *MTX* methotrexate, *IVIG* intravenous immunoglobulins

^a^Because RTX is a monoclonal antibody, administering IVIG prior to RTX may saturate the FcRn receptor, thereby precluding recycling of RTX and its sustained activity [39] and therefore administration of RTX is recommended prior to IVIG [34]

1. Rituximab (RTX) + methotrexate (MTX) + intravenous immunoglobulins (IVIG) (short course, 5 weeks)

Banugaria et al. first reported this protocol in 7 CRIM negative patients. Four patients never seroconverted, one remained with a LT of antibodies, while other two patients had a peak of 1:6400 and receiving a second course of the same ITI regimen. In fact, their protocol provided that patients with antibody titers of ≥6400 at 2 or more time-points after CD19+ B cell recovery at 5 months were administered another cycle of the ITI regimen with or without a plasma cell targeting agent [23].

Successively, Kazi et al. reported the same protocol in 19 CRIM negative patients. 15 out of 19 patients (79%) either did not develop rhGAA IgG antibodies (*n* = 8, never in ERT monotherapy group) or maintained low titers (≤6400) even after B lymphocytes reappeared. Four patients required further cycles of ITI (1 HSAT, 3 SIT) [34]. This may be due to a Fcγ receptor polymorphism which reduces the efficacy of rituximab [40]. No significant difference in baseline characteristics was noted between patients who had broken tolerance to the ERT compared with those who had maintained low/no titers. Patients who had been undergoing immunomodulation regimen, when compared to those on ERT monotherapy (*n* = 10), showed improved overall and ventilator-free survival, improved cardiomyopathy, motor function and feeding (like CRIM positive survivors on ERT), evident after the first 6 months. Conversely, during the same period of time, the monotherapy group worsened because of development of antibodies within the HSAT range. Although serious infections were noted in 4 patients, no immunomodulation regimen was withheld due to safety concerns. Titer against routine vaccines was performed after B cell recovery in 4 patients, which demonstrated the ability to mount a humoral response to other antigens, although not in all patients nor for all antigens. This data has been confirmed by other authors as well [22, 41]. Poelman et al. used the same protocol, but with a different dosage of methotrexate (Table 2) on 1 CRIM negative and 2 CRIM positive patients [36]. However, the immune regimen failed to induce immune tolerance: 2 patients developed an high and sustained titer (1 CRIM negative and 1 CRIM positive) and 1 CRIM positive patient developed a titer of 1:6250. More recently, Li et al. reviewed a cohort of CRIM negative patients, among which 20 were immunomodulated before starting ERT. All remained immunotolerant, 13 no seroconverted, 7 developed LT. Compared to historical cohort treated with ERT monotherapy (*n* = 10), of whom median peak titer was 204,800 (range 25,600-1,638,400), patients treated with ERT + ITI had significantly lower median peak titer of 0 (*n* = 20, 0–51,200). Surprisingly, within the cohort treated with ERT + ITI, although early treated patients (≤4 weeks) showed a better outcome than late treated, the number of immunotolerant patients (titre ≤6400) did not differ significantly between these groups. However, early treated patients were significantly more likely to remain seronegative than late treated patients [42].

2) Rituximab (RTX) + sirolimus or mycophenolate + intravenous immunoglobulins (IVIG)

Elder et al. described treatment of 5 infantile onset patients (4 CRIM negatives, 1 CRIM positive) using this protocol [37]. It prevented antibody formation with improved outcomes and no adverse events except in 1 CRIM negative patient (who had not received maintenance rituximab, but only mycophenolate) who developed HSAT and subsequent clinical decline. One limitation is that this protocol delays initiation of ERT for 3 weeks, which can lead to irreversible muscle damage.

### Prophylaxis protocols in CRIM positive patients

A subset of CRIM positive patients (about 40%) develop HSAT. These patients are defined as high-titer CRIM positive (HTCP patients) and have a similar pattern of antibody response and clinical decline as CRIM negative patients. The most marked difference between CRIM positive HSAT and CRIM negative patients regards the development of neutralizing antibodies which seem to be a common occurrence in CRIM negative groups. The basis for this finding is not clear; however high titers of binding antibodies such as those present in HTCP patients may also abrogate product efficacy by several mechanisms. HCTP patients have similarly poor outcomes compared to CRIM negative patients [18, 43]. The main characteristics of immune responses in different patient groups is shown in Table 3.

**Table 3:** Main characteristics of immune responses in different patient groups [18]

	CN	CP HSAT	CP LT
Median time to seroconversion	4 weeks	4 weeks	8 weeks
Median titer at 26 weeks	1:51,200	1:51200	1:400
Median titer at 52 weeks	1:153,600	1:51,200	1:200
Median peak	1:204,800	1:204,800	1:800

*CN* CRIM negative, *CP* CRIM positive, *HSAT* high sustained antibody titer, *LT* low titer

Kazi et al. used a transient low dose methotrexate protocol to prevent the developing of antibodies in naive CRIM positive patients (Table 2) [17]. In a study on 14 patients, 2 (14.2%) developed titers in the HSAT or SIT range versus 32.4% in the comparison group (*n* = 37).

A more recent work of the same group retrospectively studied the effects of a more complex protocol (short-course of rituximab, methotrexate, and/or IVIG, as described for CRIM negative patients, see above) in a cohort of 9 ERT-naive CRIM positive patients. None developed SIT or HSAT, after a median timepoint following ERT initiation of 104 weeks [38].

- **Statement #3:** On the basis of the risk-benefit ratio reported in the literature and discussion of the personal experiences of the authors, the Expert panel believes that in CRIM negative patients the first reported regimen above (RTX + MTX+ IVIG - short course, 5 weeks) has shown to have the best success in achieving long-term immune tolerance without long-term toxicity and delay in ERT initiation. In CRIM positive patients, it is possible to use the same protocol or a low-dose methotrexate protocol, because the literature data are not conclusive. Authors believe that, based on the clinical condition of the patient and the presence of other risk factor (GAA variants already reported in patients who developed SIT or HSAT, older siblings that developed SIT or HSAT, etc.), an individualized evaluation of the risk-benefit ratio is needed. In case of doubt of CRIM status, the authors agree that the patient should be treated as a CRIM negative patient until the result of the western blot analysis on fibroblasts is available.

#### Genetic predisposition to HSAT risk

An important focus is early identification of patients who are at the highest risk of developing HSAT in order to adopt more appropriate immunomodulatory regimens [18]. GAA variants alone are not reliable to predict the development of anti-ERT antibody, both in CRIM positive and in CRIM negative patients [13, 23, 25].

De Groot et al. developed an individualized T cell epitope measure (iTEM) approach for the prediction of anti-drug antibodies development. For each patient, a sum of the predicted individual T cell epitope content in rhGAA (based on the HLA-DRB1 alleles and compared to their own mutate native GAA) was calculated to determine the individual risk of developing anti-drug antibodies [44]. In their study all 5 CRIM negative subjects had a highly elevated iTEM score and, as predicted, all had high anti-drug antibody responses to ERT. Among 19 CRIM positive patients, 8/10 with high anti-drug antibodies and 7/9 with low anti-drug antibodies were correctly identified, with an overall agreement with high anti-drug antibodies of 88% (sensitivity 87%, specificity 89%). This approach personalizes risk assessment in IOPD and may be useful in identifying CRIM positive patients at high risk for HSAT/SIT development who would benefit from immunomodulation. It could be very useful in the next few years.

### Immune tolerance induction in patients who developed antibodies during ERT

The elimination of anti-ERT antibodies, particularly those within the range of HSAT, has been challenging as patients require prolonged and higher intensity immune modulation and sometimes immune suppression is unsuccessful [13, 45]. Furthermore, since antibodies take weeks to decline to clinically insignificant levels, the enzyme is less likely to have an effect during this period of HSAT and the disease continues to progress [40]. The protocols used are summarized in Table 4.

**Table 4:** Immune-tolerance induction protocols in patients who developed antibodies during ERT

**RTX, MTX, and IVIG (for patients with non-HSAT)** [46]	
• RTX: 375 mg/m^2^/dose for 4 weeks, followed by maintenance dosage every 4 to 12 weeks.	
• MTX 0.5 mg/kg weekly enterally, added after 7 weeks; IVIG 500 mg/kg every 4 weeks until antibodies are eliminated.	
**Bortezomib, RTX, MTX, and IVIG** [23, 47]	
• Bortezomib: 1.3 mg/m^2^ IV, twice weekly (day 1, 4, 8 and 11, equivalent to 1 cycle) (total 3–6 cycles).	
• RTX 375 mg/m^2^ IV (on initial round of weekly RTX infusion, thereafter RTX infusions every 4 to 12 weeks, to a maximum of 52 doses).	
• MTX 15 mg/m^2^ os; IVIG 400–500 mg/kg iv.	
**RTX, bortezomib, sirolimus, and IVIG** [48]	
• RTX 375 mg/m^2^, 3 weekly infusions.	
• Bortezomib 1.3 mg/m^2^, 6 twice-weekly doses.	
• IVIG monthly, first dose 1.0 g/kg, subsequent doses of 0.5 g/kg.	
• Sirolimus started at week 4 (10–20 kg: 1.0–1.5 mg/day; 20–30 kg: 1.5–2 mg/day; double dose on first day; dose adjusted on the basis of serum levels (reference range 4–12 μg/l).	

*ERT* enzyme replacement therapy, *RTX* rituximab, *MTX* methotrexate, *IVIG* intravenous immunoglobulins

1. Rituximab (RTX), methotrexate (MTX), and intravenous immunoglobuline (IVIG)

This protocol was shown to be effective by Mendelsohn et al. in 1 CRIM negative patient with titer 1:1600 [46], Messinger et al. in 2 CRIM negative patients with titers 1:1600 and 1:12,800 respectively [35], Markic et al. in 1 CRIM positive patient with titer 1:6400 [49], and Blasco-Alonso et al. in 1 CRIM negative patients with titer 1:32000 [50], but it often required prolonged drug administration. Moreover, the success of eliminating anti-drug antibodies is demonstrated only in patients with non-HSAT titers. It is possible that patients with HSAT may not respond to this regimen due to the use of drugs that do not target antibody producing plasma cells [43].

2) Bortezomib, rituximab (RTX), methotrexate (MTX), and intravenous immunoglobuline (IVIG)

To eliminate long-memory plasma cells, especially in HSAT patients, some authors added bortezomib, a plasma cell-targeting agent [23, 47]. These authors described 1 CRIM negative and 2 CRIM positive HSAT who after treatment showed a rapid sustained decline of the antibody titer associated with clinical improvement and reduction of urinary tetrasaccharide levels without serious adverse events or infections. Similar results were obtained by Stenger et al. in 1 CRIM positive patient with HSAT [41]. Conversely, Owens et al. reported 2 patients (1 CRIM positive and 1 CRIM negative) in whom bortezomib failed to eliminate HSAT despite the reduction in IgG antibody titer. This could be explained by the administration of a limited number of bortezomib cycles and the already advanced disease progression at the time of bortezomib initiation [51].

3) Rituximab (RTX), bortezomib, rapamycin, and intravenous immunoglobulins (IVIG)

Poelman et al. described this protocol in two CRIM negative and 1 CRIM positive patients with HSAT. The protocol did not eliminate anti-ERT antibody titers although the titers decreased and the neutralizing effects and infusion-associated reactions were no longer present. None of the patients experienced a serious adverse event [48].

Anecdotal reports have been made on the use of plasma exchange to remove pathogenic antibodies and immune complexes from plasma and high-dose IVIG (1–3 g/kg/week) in patients with adverse reactions to other drugs, but with modest results, and in patients with low antibody titers involving, in the case of plasma exchange, additional risks related to the procedure [52, 53].

Lastly, ERT dosage can have a role in determining the efficacy of the drug in patients with an antibody response: with a higher dosage (40 mg/kg/week), more antibody-free ERT should be available and the neutralizing effects of the same titer are likely to be less severe than in patients receiving 20 mg/kg every other week. For example, van Gelder et al. calculated that an ELISA titer of 1:156,250 may bind as much as 54% of the administered enzyme at a dosage of 20 mg/kg (about 10 mg/kg) [27]. Theoretically, if a similar amount is bound upon administration of 40 mg/kg, about 30 mg/kg would still be available for uptake in target tissues [27, 48]. Recently, an observational study of the European Consortium project group on 124 c-IOPD concluded that ERT dosage of 40 mg/kg/week significantly improves survival compared to standard dosage (20 mg/kg every other week) [54]. Further studies on patients with positive antibodies are needed.

- **Statement #4:** The Expert Panel believes that the best regimen in case of high antibody titer is comprised of the use of Bortezomib, Rituximab, Methotrexate and intravenous immunoglobulins. Early initiation after antibodies appear and prolonged therapy is important. Although antibody titers > 51,200 should be considered carefully, there is no antibody titer threshold to be treated. It is indeed important to consider the overall clinical picture, especially if there is clinical decline with persistent or increasing titer. In patients with sustained low titers, a dose increase may be beneficial. Furthermore, in patients who are already critically ill with a negative prognosis (e.g. artificially ventilated), discussion with parents is necessary to assess the benefit-risk of the immunotolerance induction. Additional research will be needed to establish the optimal means of tapering the maintenance regimen.

### Mechanism and adverse reactions

The immunomodulation regimens target immune cells at different levels including elimination of B cells, T cells, plasma cells and induction of GAA-specific B_reg_ and T_reg_. Table 5 summarizes the mechanisms of immune tolerance and potential adverse events of pharmaceutical agents that are commonly used. It should be noted that the duration of rituximab-induced immunosuppression is about 5 months (based on the half-life of the drug) and the increase in CD19 can be associated with increased specific antibody response [23].

- **Statement #5:** On the basis of available literature and accumulated personal experiences, the Expert Panel recommends, before starting immunomodulation, performing the following tests: blood cells count, C-reactive protein, immunoglobulins (IgA, IgG and IgM), and flow-cytometry (B and T cells) to have a baseline assessment and exclude infections. To verify the effect of rituximab on B lymphocytes, we suggest performing flow-cytometry to monitor B cell recovery (CD19% and CD20%) and total serum immunoglobulins (IgA, IgM, IgG) weekly for 2 weeks (before the next rituximab dose), and monthly thereafter (before each Ig infusion) until full B cell recovery. To monitor the side effects of methotrexate, it is useful to monitor blood counts, AST and ALT levels, and creatinine. Neutropenia and increased AST and ALT levels or fever may necessitate temporary discontinuation. Folic acid supplementation can be considered to prevent or treat MTX toxicity.

**Table 5:** Drugs commonly used for immune toleration

Drug	Mechanism	Adverse events
Bortezomib	Proteasome inhibition: blocks protein recycle and production of antibodies in plasma cells^a^ [55, 56]	Peripheral neuropathy, anemia, neutropenia, thrombocytopenia, gastrointestinal and cardiac side effects [23]
IVIG	Binding to the neonatal Fc receptor (FcRn) which is responsible for recycling of antibodies thus downregulating antibody responses [53, 57, 58] Passive immunity during the period of immune suppression due to other drugs (specially rituximab) [23]	Infusion-associated reactions [59]
Methotrexate	Inhibits folic acid metabolism (which blocks de novo DNA synthesis), thus eliminating dividing B and T cells. Low dose: Induces regulatory B cells rather than cell depletion [60, 61]	Bone marrow and gastrointestinal toxicities, rarely acute pneumonitis, pulmonary fibrosis and renal function impairment [12, 62]
Mycophenolate mofetil	Inhibition of proliferative responses of T and B lymphocytes [12]	Leukopenia, anemia and thrombocytopenia
Rapamycin	Mammalian target of rapamycin (mTOR), which inhibits cell survival and proliferation of B and T lymphocytes, but selectively promotes regulatory T – Treg – cells ^b^ [63–67]	
Rituximab	Monoclonal antibody against CD20 molecule expressed on B cells [12]	Infusion-associated reactions, lymphocytopenia, progressive multifocal leukoencephalopathy [62]

^a^ In muscle, the ubiquitin-proteasome system is believed to degrade contractile skeletal muscle proteins and may play a critical role in muscle wasting [23]

^b^ May have an impact on glycogen storage in muscle by influencing the mTor pathway and inhibiting glycogen storage [68]

Immunomodulation regimens are relatively non-antigen specific and have the potential to cause generalized immune suppression with a risk of infection, also with opportunistic pathogens, and malignancy [23, 37]. Patients with Pompe disease are already susceptible to lung involvement with higher risk of infections. Surprisingly, very few reports of severe life-threatening infections have been reported. This may be due to the use of intravenous immunoglobulins [23] and to the improvement of the underlying disease [12]. There are anecdotal reports of the use of preventive antibiotic therapy (e.g. azithromycin) during immunomodulation regimens [48]. Vaccination response might be diminished during immunotolerance induction and during the following 6 months [23]. There is little documented experience on the long-term effects of immune modulating therapies in children over time and long-term assessment is needed.

- **Statement #6:** Our Expert Panel recommends observing adequate hygiene rules during the immunomodulation regimen and to avoid closed and crowded places and sources of infection. The authors believe that preventive antibiotic therapy is not routinely required. In cases of fever, prompt medical evaluation is recommended, and in cases of signs or symptoms suggestive of bacterial infection, antibiotic therapy must be started promptly. Live vaccines should be avoided while on treatment and immediately following treatment until there is full B cell recovery. Non-live vaccines can be given according to the vaccination schedule, but vaccination response should be monitored even after B cell recovery and, in case of inadequate response, additional vaccine boosts should be considered. During flu season, it is recommended to vaccinate all close contacts of the subject.

### Hypersensitivity reactions to ERT

About half of patients with Pompe disease experience infusion associated reactions which can involve a wide range of clinical symptoms especially cardiac, respiratory, cutaneous and/or gastrointestinal manifestations. Approximately 1% of patients develop anaphylactic shock and/or cardiac arrest that requires life-support measures, while 5–14% of patients develop significant allergic reactions that involve at least 2 or 3 body systems [5]. These can appear at various times, even years after the start of the ERT [27]. Risk factors include the presence of IgE (which does not seem to correlate with IgG), acute illness at the time of the infusion, or a very high-rate infusion regimen, while there does not seem to be a correlation with dosage or IgG levels [22, 69, 70]. In a previous Italian observational study, among 128 patients eight experienced hypersensitivity reactions (6.25%) [28].

Hypersensitivity reactions can be classified as allergic and non-allergic. Allergic hypersensitivity, either IgE- or non-IgE mediated, is antigen specific. In this case skin tests and intradermal tests are usually positive and tryptase increases within 1 h after clinical reaction (peaks at 60–90 min after the onset of symptoms and remain elevated for up to 5 h), as the reaction is mast cell/basophil mediated [71–73]. A non-allergic reaction (more frequent) is not antigen specific. An immunologic pathogenic mechanism has not been demonstrated, but may be related to mechanisms of complement activation, cytokine release, and direct mast cell stimulation [5].

As infusion-associated reactions of various mechanisms have similar presentation, clinicians are unable to use clinical history to predict these events and guide management [74]. After a potential hypersensitivity reaction has been identified, three approaches are possible: standard prevention and management, immunomodulation, and desensitization [75].

*Standard prevention and management include*: slowing down or interrupting infusion and then restored it at the last safe rate (then, gradually increasing dose, rate, and concentration), monitoring of vital signs, symptom-specific medical intervention (e.g. antipyretics, antihistamines, corticosteroids, inhaled short-acting beta2-agonists if bronchoconstriction, epinephrine if anaphylaxis) and drawing blood for laboratory tests [3, 10, 11, 76–78]. After an adverse reaction, a premedication protocol can be applied. Most of the premedication protocols provide for the use of antihistamines, glucocorticoids, and antipyretics [79]. However, prophylactic antihistamines are not recommended in patients with a history of IgE positive hypersensitivity reactions, as this may mask early symptoms of a hypersensitivity reaction such as a cutaneous reaction [69]. Some authors have suggested the use of tranexamic acid (500 mg/day), especially in patients with angioedema or complement-mediated infusion-associated reactions, since it inhibits plasmin activation of kallikrein and interrupts the kinin generating cycle [79]. A novel approach for IgE mediated anaphylaxis uses an IgE monoclonal antibody, namely omalizumab. One patient had been successfully treated with omalizumab, but he appeared to require its chronic administration to continue ERT. Interestingly, this patient, who was CRIM negative, also maintained a low IgG antibody titer suggesting that omalizumab, perhaps via signaling through the Fcε receptor, had also limited development of the IgG response [80]. Nonetheless, further studies are needed.

- **Statement #7:** In patients at high risk (e.g. acute illness at the time of the infusion) ERT should be administered at a slower rate. After a reaction, slowing or interrupting infusion and supportive measures are recommended. It is useful to draw blood for analysis of IgE titer, tryptase and complement activation. Our Expert Panel believes that a routine premedication is not particularly useful, but after an infusion associated reaction a premedication with 4 drugs is recommended: antihistamines (H2 antagonist + H1 antagonist), unless specific IgE antibodies are present, glucocorticoids and tranexamic acid if angioedema or complement activation are present.

Some *immunomodulation approaches*, as those described above, have also demonstrated to alleviate hypersensitivity reactions, if it is sustained by an antibody response. Thus, they are a limited role in cases with positive anti-rhGAA antibodies. Moreover, these regimes result in significant risks and reappearance of antibodies following successful initial suppression has been reported [13, 78].

If these measures fail or are unapplicable, *desensitization* is strongly indicated. Desensitization is the induction of a temporary state of unresponsiveness to a compound responsible for a hypersensitivity reaction (IgE and non-IgE mediated) by increasing sub-therapeutic doses over a short period of time until the total cumulative therapeutic dose is delivered. It is a high-risk procedure and should be used only in patients in whom there is no safe, effective alternative drug treatment (e.g. ERT in Pompe disease) and after careful risk-benefit analysis [72].

Infants with Pompe disease subject to a desensitization protocol require attention to special aspects such as the need for administration of a large amount of enzyme (compared to patients on ERT for other disorders), and the restricted amount of fluid intake allowed due to underlying cardiomyopathy [69]. Particular attention should be paid to patients at increased risk because of a comorbidity such as hemodynamically unstable patients, those with uncontrolled cardiac disease, or subjects who have experienced severe anaphylaxis. No specific guidelines yet exist. General rules are suggested by the “EAACI consensus statement on rapid desensitization for drug hypersensitivity” [61]. Any concomitant medication must be continued however drugs such as beta-blockers, which can interfere with the treatment of a severe hypersensitivity reaction, should be discontinued whenever possible. Generally it is preferable to use desensitization protocols after they have been successfully proven on at least 10 patients [61] although only a few Pompe patients having ERT with desensitization have been described.

To our knowledge, 10 cases (8 pediatric, 2 adults) affected by Pompe disease and subjected to desensitization protocol have been reported [14, 56, 74, 75, 79, 81, 82] (Table 6). Of note, recently Emecen Sanli et al. reported the first case of successful concomitant immunotolerance induction and desensitization protocol in a CRIM negative 7-month-old male patient, that have developed IARs and anti-rhGAA antibodies (1:12,800) [83].

**Table 6:** Summary of patients undergoing desensitization for drug hypersensitivity

Sex/age	Phenotype/CRIM status	Symptoms	Immune reaction	Premedication	Desensitization: initial concentration	Desensitization: total dose	Desensitization: adverse reactions
M, 11 months [79]	IOPD CP	Urticarial rash	Elevated serum tryptase, activated complement, IgE and eosinophil count normal	Tranexamic acid, deflazacort, cetirizine, ranitidine	0.5 μg/ml	10 mg/kg (only for the first infusion)	minimal skin reactions
M, 4 months [80]	IOPD CP	Anaphylaxis	IgE and eosinophil count normal	Tranexamic acid, deflazacort, cetirizine, ranitidine	15 μg/ml	Full dose 20 mg/kg EOW	NO
F, 4 months [82]	IOPD, CRIM ND	Urticarial rash	intradermal test positive, SPT negative	Diphenhydramine, methylprednisolone	0.1 μg/ml	20 mg/kg EOW	NO
M, 3 years 8 months [69]	Juvenile onset PD CP	Anaphylaxis	IgG 1:51200, negative IgE titers, complement activation, normal serum tryptase	Diphenhydramine, acetaminophen	0.07 μg/ml	10 mg/kg/week	severe reactions (requiring epinephrine)
M, 10 months [69]	IOPD CP	Urticarial rash	Elevated IgE and serum tryptase, negative IgG and no complement activation	No	0.05 μg/ml	10 mg/kg/weekly	minor skin reaction
F, 6 years 9 months [74]	IOPD CP	Urticarial rash	SPT negative, IgG negative	Prednisolone	0.01 μg/ml	10 mg/kg/weekly	Urticarial rash
F, 15 months [75]	IOPD CP	Anaphylaxis	IgG 1:6400, normal IgE, tryptase and complement	No	1:100	0.2 mg/kg. The dose was doubled every week	No
F 7 months [82]	IOPD CP	Urticarial rash, intractable cough and stridor	Intradermal test positive, SPT negative	No	0.05 μg/ml	10 mg/kg/week	Urticarial rash
F 28 y [56]	LOPD	Anaphylaxis	IgE positive, IgG 1600, SPT negative, intradermal testing positive, complement testing negative, elevated tryptase levels	Diphenhydramine and prednisone	0.1 μg/ml	10 mg/kg/week	severe reactions (requiring epinephrine)
F, 46 years [56]	LOPD	Anaphylaxis	SPT and intradermal tests negative, normal IgE, eosinophil counts, complement, tryptase	Antihistamines H1 (cetirizine), antileukotriene (montelukast)	Drop by drop of ERT solution 2 mg/ml with increasing flow + saline solution with continuous flow by another perfusion pocket	Full dose	Minor reaction: anxiety, crying, abdominal pain

*IOPD* infantile onset Pompe disease, *LOPD* late onset Pompe disease, *CP* CRIM positive, *CN* CRIM negative, *ND* not determined, *SPT* skin prick test, *EOW* every other week

Most of these protocols (6/10) administered a half dose (10 mg/kg) once a week. Only one patient was on a very low dose of ERT for a longer period (8 weeks) [75]. Breakthrough reactions are drug hypersensitivity reactions that occur despite the desensitization procedure. These most often occur during the first course of desensitization [72]. Such reactions have been reported in 7 of 10 patients and were mild/moderate in 4, while 3 patients experienced severe infusion-associated reactions including anaphylactic reactions requiring epinephrine injections in 2 cases (Table 6). After a breakthrough reaction, some authors did not modify the protocol, while others decelerated the dose escalation introducing intermediate dosing steps, or going back one or two steps, depending, however, on the severity of the reaction.

- **Statement #8:** If other measures fail, desensitization is recommended. The infusion should be carried out with micro dilutions, adjusting the dose/rate based on the patient’s clinical manifestations and tolerance. In particular, protocols with a significant dose reduction should be avoided in patients with severe symptoms of the disease such as cardiomyopathy, as prognosis can be compromised in case of transient worsening. On the basis of the literature, a proposal of a desensitization protocol is reported in Table 7.

**Table 7:** Proposal of desensitisation protocol

Initial dose	10 mg/kg weekly
Dose increase [69]	¾ of the dose every 10 days (after 2 successive successfully infusions) → full dose at a modified rate scheduled every 2 weeks → standard recommended rate
Initial concentration [72]	1/10,000–1/100 of the full therapeutic dose. If anaphylaxis is severe: 1/1,000,000–1/10,000 of the full therapeutic dose. If positive skin test, the starting dose can be determined based on the endpoint titration
Concentration increase [74]	Doubling or three-fold every 15–20 min until the therapeutic dose is achieved

## Conclusions and future prospective

Herein a summary of the evidence on adverse reactions to ERT in Pompe disease, particularly in its c-IOPD, is presented. Based upon our review of the available literature, we can conclude that there remains a need for the following.

- A novel approach to immune tolerance induction, especially protocols that are antigen-specific and/or more highly antigen targeted, rather than employing systemically immunosuppressive agents, in order to improve efficacy and safety.
- More personalized treatment approaches including immunogenicity prediction prior to ERT initiation, especially in CRIM positive patients.
- Further strategies for eliminating long-lived plasma cells.
- ERT Engineering to create non-immunogenic epitopes without reducing effectiveness.

In the future, gene therapy may play a relevant role by providing low doses of a non-toxic viral vector associated with ERT which could minimize the immune response.
